# Accidental falls in middle-aged women

**DOI:** 10.11606/s1518-8787.2020054002579

**Published:** 2020-12-04

**Authors:** Lígia Raquel Ortiz Gomes Stolt, Daniel Vieira Kolish, Maria Regina Alves Cardoso, Clarice Tanaka, Erika Flauzino Silva Vasconcelos, Elaine Cristina Pereira, Máyra Cecilia Dellú, Wendry Maria Paixão Pereira, José Mendes Aldrighi, Ana Carolina Basso Schmitt

**Affiliations:** I Universidade Federal da Paraíba Departamento de Fisioterapia João PessoaPB Brasil Universidade Federal da Paraíba. Departamento de Fisioterapia. João Pessoa, PB, Brasil; II Universidade de São Paulo Faculdade de Medicina Programa de Pós-Graduação em Ciências da Reabilitação São PauloSP Brasil Universidade de São Paulo. Faculdade de Medicina. Programa de Pós-Graduação em Ciências da Reabilitação. São Paulo, SP, Brasil; III Articulab – Ortopedia Moderna Especializada São PauloSP Brasil Articulab – Ortopedia Moderna Especializada. Fisioterapeuta em reabilitação ortopédica e facilitador de processos de trabalho e desenvolvimento de projetos. São Paulo, SP, Brasil.; IV Universidade de São Paulo Faculdade de Saúde Pública Departamento de Epidemiologia São PauloSP Brasil Universidade de São Paulo. Faculdade de Saúde Pública. Departamento de Epidemiologia. São Paulo, SP, Brasil; V Universidade de São Paulo Faculdade de Medicina Departamento de Fisioterapia, Fonoaudiologia e Terapia Ocupacional São PauloSP Brasil Universidade de São Paulo. Faculdade de Medicina. Departamento de Fisioterapia, Fonoaudiologia e Terapia Ocupacional. São Paulo, SP, Brasil; VI Centro Universitário FUNVIC PindamonhangabaSP Brasil Centro Universitário FUNVIC. Curso de Fisioterapia. Pindamonhangaba, SP, Brasil; VII Universidade de Taubaté Departamento de Fisioterapia TaubatéSP Brasil Universidade de Taubaté. Departamento de Fisioterapia. Taubaté, SP, Brasil; VIII Universidade de São Paulo Faculdade de Saúde Pública Departamento de Saúde Materno-Infantil São PauloSP Brasil Universidade de São Paulo. Faculdade de Saúde Pública. Departamento de Saúde Materno-Infantil. São Paulo, SP, Brasil; IX Universidade de São Paulo Faculdade de Medicina Hospital das Clínicas São PauloSP Brasil Universidade de São Paulo. Faculdade de Medicina. Hospital das Clínicas. Laboratório de Investigação em Fisioterapia. São Paulo, SP, Brasil

**Keywords:** Women, Fall Accidents, External Causes, Epidemiology, Prevalence

## Abstract

**OBJECTIVE:**

To estimate the prevalence of accidental falls in women and to identify possible associations of sociodemographic, clinical and lifestyle variables with falls, in 2007 and 2014.

**METHODS:**

Two cross-sectional studies were performed, in 2007 and 2014, within the *Projeto de Saúde de Pindamonhangaba* (PROSAPIN – Pindamonhangaba Health Project), with women aged between 35 to 75 years. Probabilistic samples were selected among women living in the municipality and participating in the Health Family Strategy. Data collection included: face-to-face interview, anthropometric examination and blood test. The outcome variable “have you fallen in the last six months?” was raised during the interview. The prevalence of falls in 2007 and 2014 were estimated by score with a 95% confidence interval (95%CI). Multiple logistic regression models were constructed to identify the association of independent variables with the occurrence of falls for each year based on the odds ratio (OR). We used the Stata 14.0 software for statistical analysis.

**RESULTS:**

The prevalence of accidental falls were: 17.6% (95%CI 14.9–20.5) in 2007 and 17.2% (95%CI 14.8–19.8) in 2014. In 2007, factors associated with falls were: aged 50–64 years (OR = 1.81; 95%CI 1.17–2.80), high school (OR = 1.76; 95%CI 1.06–2.93), hyperuricemia (OR = 3.74; 95%CI 2.17–6.44), depression (OR = 2.07; 95%CI 1.31–3.27), poor sleep (OR = 1.78; 95%CI 1.12–2.82) and daytime sleepiness (OR = 1.86; 95%CI 1.16–2.99). In 2014, they were: aged 50–64 years (OR = 1.64; 95%CI 1.04–2.58), hyperuricemia (OR = 1.91; 95%CI 1.07–3.43) and depression (OR = 1.56; 95%CI 1.02–2.38), plus metabolic syndrome (OR = 1.60; 95%CI 1.03–2.47) and musculoskeletal pain (OR = 1.81; 95%CI 1.03–3.18).

**CONCLUSIONS:**

Falls occur significantly in women aged 50 years or over, indicating that they are not restricted to older adults and that there is a need to initiate preventive measures earlier. Both studies showed similar magnitudes of occurrence of accidental falls and reinforced their multifactorial nature. In addition, hyperuricemia may be a potential new factor associated with falls.

## INTRODUCTION

Accidental falls are unexpected events in which people collapse to the floor or to a lower level^1 , 2^ . Its causes are multifactorial, and the occurrence of falls in older adults has been associated with: being a woman^3^ , almost falling^6^ , advanced age^5^ , vision impairments^3 , 7 , 8^ , urinary incontinence^6^ , cardiovascular diseases^6^ , obesity^7^ , sleep disorders^3 , 4 , 6^ , diabetes^6^ , depression^6^ , among others.

Fall accidents have been the subject of constant study in the scientific community, due to their relevance as a health problem characteristic of elderly people^9^ . They affect about 30% of elderly people^2 , 4^ , being the main cause of accidental injuries in the United States^9^ and occupying the third position among the causes of accidental deaths both in the United States^9^ and in Brazil^10^ .

This type of problem has been extensively studied in older people^6 , 8 , 11^ . However, few and recent international studies indicate that falls injure adults of all ages^9 , 12^ — 35.3% of those who suffer these injures are middle-aged^9^ . In Brazil, we found only one study^5^ dedicated to the topic, raising the need to fill this knowledge gap, in order to deepen and expand studies related to accidental falls in younger populations, including middle-aged people.

Some current studies point out that the prevalence of falls varies in populations, and is higher among Caucasians than in Chinese^13^ and characteristically lower in Eastern older people^3 , 9^ , arousing the interest of researching countries where the population is predominantly mixed and multicultural, such as Brazil.

Falls are more common in females^3 - 5^ and have a variable frequency according to geographical region and age. This is evident when comparing the results of a study conducted in Australia^4^ , in which 32.2% of female older adults fell, with another study conducted in China, in which 19.4% of women aged over 45 years suffered falls, and with a third study, performed in Brazil, which found that 30.8% of women aged over 55 years had suffered accidental falls^5^ . It is also noteworthy that adult women go through a significant hormonal event in their lives, menopause^7^ , with consequent osteometabolic disorders, such as osteoporosis. Older women are the ones who suffer the most injuries from falls^14^ , and, among these injuries, fractures represent 28% of the total^9^ .

Given this, our study aimed to estimate the prevalence of accidental falls in women aged 35 to 75 years, in addition to identify possible associations of sociodemographic, clinical and lifestyle variables with falls, in 2007 and 2014.

## METHODS

### Population and sample

In 2007 and 2014, two cross-sectional studies were performed within the *Projeto de Saúde de Pindamonhangaba* (PROSAPIN – Pindamonhangaba Health Project) with women aged 35 to 65 years (in 2007) and 35 to 75 years (in 2014), participants in the Family Health Strategy (FHS). In 2007, the primary health care network of Pindamonhangaba/SP comprised 18 FHS units, with population coverage of 45,537 individuals. In 2014, it expanded to 21 units, covering 57,852 people.

The sample was probabilistic, using the systematic sampling procedure, stratified by age of the women and health unit, with proportional sharing. The sample size was calculated from the lowest estimated prevalence among the clinical conditions of interest in the study. Considering the prevalence of diabetes in Brazilian women in the age group of interest (9.7%)^15^ , a maximum error of 3% in 95% of the possible samples and also adjusting for possible losses (20%), the final sample for 2007 was estimated at 875 women, and for 2014, 1,200 women.

The reference population was composed of women living in Pindamonhangaba, in the age groups specified in 2007 and 2014, registered in the FHS. We excluded from the study those outside the territory covered by the FHS or who had physical and/or mental inability to participate in the collection, in addition to pregnant and deceased women. The study was approved by the Research Ethics Committee of the Faculdade de Saúde Pública of the Universidade de São Paulo/Brazil, Protocol No. 1776 (2007) and No. 312.957 (2014). All participants signed an informed consent form before their inclusion in the study.

### Data Collection

Data collection was performed in three stages: 1) obtaining information via questionnaire applied in the face-to-face interview, addressing the dependent variable and the sociodemographic, clinical and lifestyle variables; 2) physical examination and anthropometry, performed by trained and calibrated researchers; and 3) blood analysis examination.

### Outcome Variable

The outcome variable was the occurrence of falls, investigated during the interview with the question: “Have you fallen in the last six months?” If so, they were asked “How many times?”. The occurrence of a single fall characterized the woman as a faller. Falls were detailed according to circumstances (gait, transfer), causes (intrinsic, extrinsic) and consequences (help to get up after the fall, need for hospitalization, occurrence and location of post-fall fracture, and use of gait aids).

### Sociodemographic Variables

During the interview, we investigated the age of the person, which was subsequently stratified in three categories – 35 to 49 years, adults; 50 to 64, middle-aged; 65 to 75 years, older adults –, with cut-off points aiming at comparison with other studies. The self-declared color or race was determined as white, black, yellow, brown or indigenous. Marital status was self-reported as married, separated, divorced, widowed or single. Schooling was considered from the highest degree of completed education (literate, elementary school, high school, Bachelor’s degree or postgraduate studies, adult education: supplementary and literacy). We also investigated their remunerated activity or occupation: work as economic activity or occupation remunerated in cash, products, goods or benefits for at least one hour a week (exercises or does not exercise a remunerated activity). Finally, the type of FHS unit was collected, classified in rural, urban or mixed by the National Register of Health Establishments.

### Clinical Variables

Self-reports of menopause, osteopenia or osteoporosis, heart disease and hyperuricemia were investigated during the interview. For depression, the validated Beck Depression Inventory^16^ was used (score: 0 to 9 = absence or minimal symptoms; 10 to 18 = mild depression or dysphoria; 19 to 29 = moderate depression; 30 or more = severe depression). Musculoskeletal pain was surveyed through the Nordic Questionnaire^17^ . Metabolic syndrome was considered according to the International Diabetes Federation^18^ , and it was necessary to have three of five items, necessarily including the first: 1) waist > 80cm; 2) blood pressure > 130 mmHg for systolic, or 85 mmHg for diastolic; 3) high density cholesterol < 50 mg/dL; 4) triglycerides > 150 mg/dL; and 5) fasting glucose > 100 mg/dL. The presence of fasting glucose greater than or equal to 126 mg/dL, or self-reported diabetes associated with hypoglycemic medication, characterized diabetes^18^ . Blood pressure was measured, according to the 7th Brazilian Guideline for Hypertension^19^ , using a calibrated, digital, automatic, tested and validated MicroLife arm sphygmomanometer. Three blood pressure measurements were performed in the left arm, with one-minute intervals, with the participant sitting. The mean of two values was obtained, discarding the discrepant. We considered as hypertensive women with systolic blood pressure greater than or equal to 140 mmHg, or diastolic greater than or equal to 90 mmHg^19^ , or those who declared themselves hypertensive and used antihypertensive medication.

### Lifestyle variables

Sleep disorders were investigated: the Pittsburgh Sleep Quality Index^20^ evaluated the quality of sleep in the last month, divided in seven domains (score from 0 to 21; if greater than 5, sleep is considered poor); the Epworth Sleepiness Scale^21^ investigated the presence of excessive daytime sleepiness and measured the possibility of dozing off (score from 0 to 24; if greater than 10, there is excessive daytime sleepiness).

As quality control, field coordinators were present at the collection sites, and about 10% of the interviews were partially via telephone.

### Data Analysis

The prevalence of falls in 2007 and 2014 were estimated by score with a 95% confidence interval (95%CI). To identify the factors associated to falls, we used simple and multiple logistical regressive models, estimating the odd ratio (OR) with their respective 95%CI. Multiple models were built including, initially, all the variables that appeared as associated in the simple analyses, then, all the other variables were tested. Statistical calculations were performed using the Stata 14.0 software.

## RESULTS

**Figure 1:**
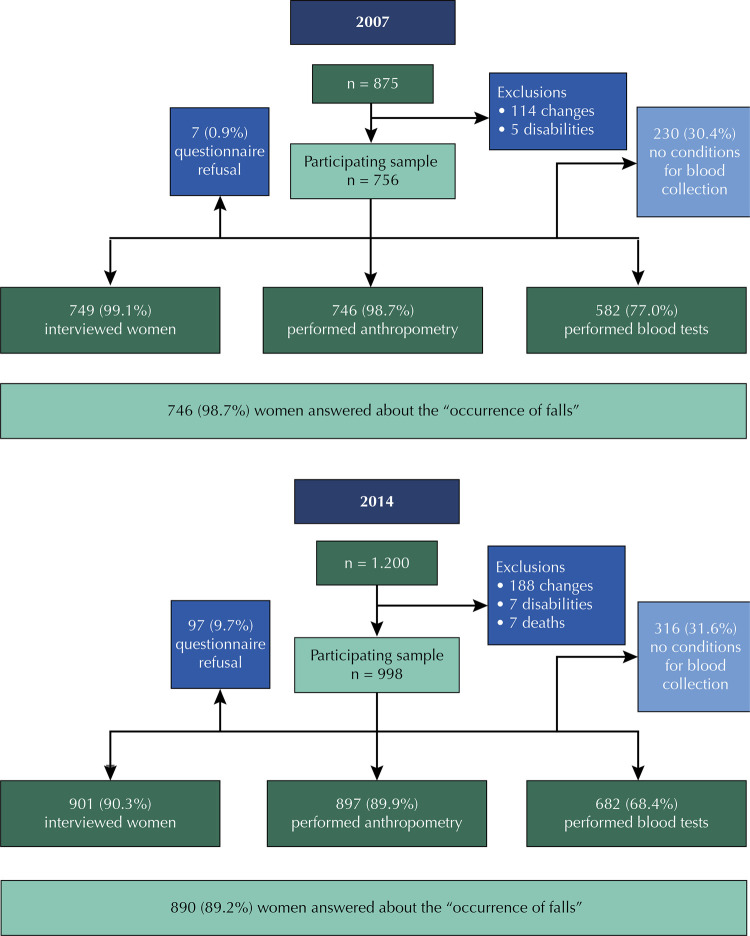
Estimated sample participating in the PROSAPIN in 2007 and 2014.

A total of 756 women participated in the study in 2007, and 998 in 2014 ( Figure 1 ). In 2007, the mean age was 47.7 years (standard deviation (SD) of 8 years) and, in 2014, 51.9 (SD – 8.8). In 2007, the sample was composed of women aged 35–65 years, of whom only one was 65 years old. In 2014, there were 13 women in this age group, some of whom participated from the beginning of the study, and others who were included in 2014.

Prevalence (95%CI): 2007: 17.6% (14.9–20.5); 2014: 17.2% (14.8 – 19.8).

Most of them went to school, had a partner and lived in neighborhoods served by urban primary health care units. About half of the women declared themselves as white, and the same amount stated that they were engaged in a paid activity.

**Figure 2:**
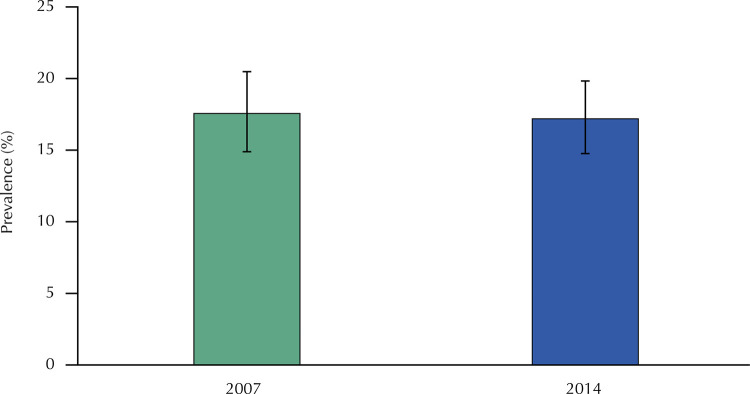
Prevalence of fall accidents in women of the PROSAPIN in 2007 and 2014.

**Figure 3:**
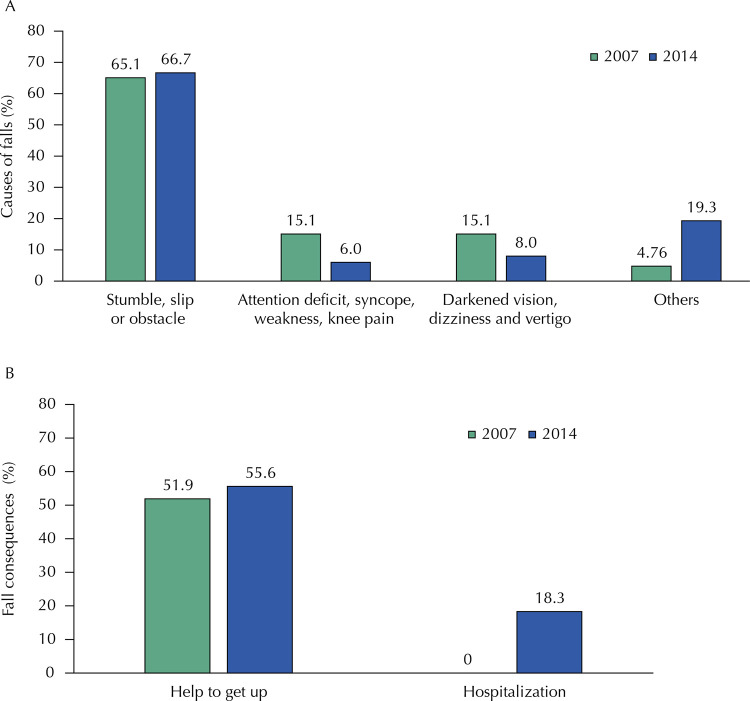
Causes (A) and consequences (B) of fall accidents in women of the PROSAPIN in 2007 and 2014.

The prevalence of accidental falls was similar in the two moments studied ( Figure 2 ). A total of 54.8% of women in 2007, and 72.4% in 2014, had a single fall, which occurred mainly while walking (52.7% and 64.2%, respectively). The main motivators of falls were stumbles, slips and obstacles. Worryingly, most needed help to get up, and, in 2014, about 18% of them needed to be hospitalized ( Figure 3 ). Fractures occurred in about 12% of the cases, mainly in the wrist (7.63% in 2007, and 4.64% in 2014), and only eight people (three in 2007 and five in 2014) used gait aids.

The simple regression analysis preceded the multiple regression analysis and did not establish associations of the variables menopause (OR = 1.66; 95%CI 1.13–2.43 / OR = 1.60; 95%CI 1.10–2.33), osteopenia and osteoporosis (OR = 0.96; 95%CI 0.29–3.26 / OR = 1.10; 95%CI 0.48–2.51), heart diseases (OR = 1.66; 95%CI 0.96–2.87 / OR = 1.76; 95%CI 1.10–2.81), diabetes (OR = 1.85; 95%CI 1.08–3.17 / OR = 1.39; 95%CI 0.92–2.10) and hypertension (OR = 1.29; 95%CI 0.88–1.89 / OR = 1.39; 95%CI 0.98–1.97) with accidental falls, in 2007 and 2014. In the multiple regression analyses, the models were distinct for 2007 ( Table 1 ) and 2014 ( Table 2 ). When observing the net weight of each variable, hyperuricemia stands out in the two analyzed moments. Age (50-64 years), hyperuricemia and depression were associated with accidental falls both in 2007 and 2014. Sleep disorders and education (high school) were important only in 2007, while musculoskeletal pain and metabolic syndrome appeared as associated with falls only in 2014.

**Table 1:** Results of the simple (OR) and multiple (adjusted OR) regression models for accidental falls and sociodemographic, clinical and lifestyle variables, PROSAPIN 2007.

Sociodemographic, clinical and lifestyle variables	n (%)	OR 2007			Adjusted OR 2007		
		OR	95%CI	p	OR	95%CI	p
**Age** ^**a**^ **(years):**							
30–49	62 (13.8)	Ref.	-	-	Ref.	-	-
**50–64**	68 (23.8)	**1.94**	1.32–2.84	**0.001**	**1.81**	1.17–2.80	**0.008**
65–75	1 (9.1)	0.62	0.07–4.94	0.654	-	-	-
**Education** ^**b**^							
Literacy, elementary or adult education	86 (15.9)	Ref.	-	-	Ref.	-	-
**High school**	30 (20.1)	1.33	0.83–2.11	0.225	**1.76**	1.06–2.93	**0.029**
Bachelor’s degree or postgraduate studies	6 (42.9)	**3.95**	1.34–11.70	**0.013**	-	-	-
**Hyperuricemia**	33 (41.3)	**3.90**	2.37–6.40	**<0.001**	**3.74**	2.17–6.44	**< 0.001**
**Depression**	69 (27.4)	**2.63**	1.79–3.86	**<0.001**	**2.07**	1.31–3.27	**0.002**
Metabolic syndrome	54 (20.1)	**1.72**	1.06–2.78	**0.027**	-	-	-
**Poor sleep**	83 (24.7)	**2.47**	1.67–3.65	**<0.001**	**1.78**	1.12–2.82	**0.014**
**Daytime sleepiness**	45 (26.2)	**2.01**	1.33–3.02	**0.001**	**1.86**	1.16–2.99	**0.010**

OR: odds ratio; 95%CI: 95% confidence interval.

Note: Statistical significant values are shown in bold.

Ref.: Reference categories for:

^a^ Age (years): Reference category = (30–49).

^b^ Education: Reference category = literate, elementary or adult education.

Hyperuricemia, depression, poor sleep, daytime sleepiness, metabolic syndrome and musculoskeletal pain: reference category = absent.

**Table 2:** Results of the simple (OR) and multiple (adjusted OR) regression models for accidental falls and sociodemographic, clinical and lifestyle variables, PROSAPIN 2014.

Sociodemographic, clinical and lifestyle variables	n (%)	OR 2014			Adjusted OR 2014		
		OR	95%CI	p	OR	95%CI	p
**Age** ^**a**^ **(years)**							
30–49	48 (12.8)	Ref.	-	-	Ref.	-	-
**50–64**	92 (20.7)	**1.79**	1.22– 2.61	**0.003**	**1.64**	1.04– 2.58	**0.033**
65–75	13 (18.6)	1.56	0.79–3.06	0.197	-	-	-
**Education** ^**b**^							
Literacy, elementary or adult education	109 (18.4)	Ref.	-	-	-	-	-
High school	22 (11.1)	**0.55**	0.33– 0.90	**0.017**	-	-	-
Bachelor’s degree or postgraduate studies	9 (18.8)	1.02	0.48– 2.16	0.958	-	-	-
**Hyperuricemia**	26 (28.9)	**2.18**	1.33– 3.57	**0.002**	**1.91**	1.07– 3.43	**0.030**
**Depression**	92 (20.9)	**1.69**	1.18– 2.40	**0.004**	**1.56**	1.02– 2.38	**0.042**
**Metabolic syndrome**	81 (21.9)	**1.78**	1.18– 2.69	**0.006**	**1.60**	1.03– 2.47	**0.035**
**Musculoskeletal pain**	127 (19.0)	**1.77**	1.13– 2.78	**0.013**	**1.81**	1.03– 3.18	**0.040**
Poor sleep	46 (18.5)	1.13	0.77– 1.65	0.527	-	-	-
Daytime sleepiness	37 (17.3)	1.00	0.67– 1.51	0.965	-	-	-

OR: odds ratio; 95%CI: 95% confidence interval.

Note: Statistical significant values are shown in bold.

Ref.: Reference categories for:

^a^ Age (years): Category of reference = (30–49).

^b^ Education: Reference category = literate, elementary or adult education.

Hyperuricemia, depression, poor sleep, daytime sleepiness, metabolic syndrome and musculoskeletal pain: reference category = absent.

## DISCUSSION

The estimated prevalence of accidental falls in 2007 and 2014 were similar, and are within the range of a study performed with middle-aged people (8.7-31.1%) from Australia, Netherlands, Ireland and Great Britain^12^ . But, when compared with studies involving older adults in Australia^4^ (32.2%), England^8^ (27.3%) and even Brazil^11^ (29.1-32.7%), they are lower, which facilitates the understanding that there is a higher prevalence of falls at older ages.

However, when analyzing the 2007 and 2014 data on accidental falls according to age group, it was found that the highest prevalence occurred in middle-aged women (50-64 years), with respective values of 23.8% and 20.7%. These, contrary to what was expected, were higher than the estimated prevalence for elderly women (65-75 years), possibly due to the small number of women in this age group. On the other hand, they are similar to the prevalence found for Thai^22^ and Chinese^3^ older men. It is also emphasized that, if women begin to fall at an earlier age, they have a greater chance of falling again in the years to come, becoming recurrent fallers. Unfortunately, there is an expectation of more serious consequences in recurrent falls, resulting in increased hospitalizations and deaths^13^ .

Falls bring overwhelming consequences, causing about 80% of disabilities due to unintentional injuries in adults over the age of 50 years^14^ in Mexico, Ghana, India, Russia and South Africa — that is, they are not exclusive to older adults.

It is worth mentioning that falls cause specific fractures according to the age group^23^ . Older adults often fall at low speed and on the hip, increasing the risk of fractures at the site. Middle-aged adults move at greater speed and fall on their arms, fracturing in particular the humerus or distal forearm (wrist)^23^ . The fact that most of the fractures occurred in this study were in the wrist corresponds to the characteristics of fractures in middle-aged people.

Most factors associated with falls of middle-aged women are also associated with falls of older adults. There were changes in these factors in the composition of the multivariate regression models between 2007 and 2014: three of them (50-64 years, hyperuricemia and depression) persisted in the composition; poor sleep, daytime sleepiness and education (high school) were specific for 2007, and metabolic syndrome and musculoskeletal pain, for 2014.

Among the factors associated with the occurrence of falls in adult women, age^5^ , depression^7 , 8^ , metabolic syndrome^22^ , musculoskeletal pain^7 , 24 , 25^ , poor sleep^3 , 5^ and excessive daytime sleepiness^4^ have already been associated with the occurrence of falls in older adults, and in people in other age groups as well.

Advanced age is often associated with falls in both sexes^8^ . However, there was a significant association between the age of 50–64 years with the occurrence of falls, revealing that there was a higher prevalence in middle-aged women than in younger adults. However, accidental falls do not constitute health problems exclusive to older adults, despite the large quantity of studies performed with this population.

The occurrence of falls in middle age and therefore, before senescence, has been pointed in some recent studies^7 , 9^ , which recorded a high incidence (42.8%, 95%CI 34.9–50.8) of women in this age group. One study^12^ reinforces the occurrence of falls in middle-aged people, especially in women. In Brazil, a single study conducted with people aged 55 years and over^5^ found an association between falls and advanced age (equal or over 65 years).

Therefore, we found that women aged 50 to 64 years, still in middle age, fall significantly, breaking the paradigm of accidental falls being restricted to older adults — a fact not yet published in national studies. In addition, it is evident that falls are striking in the life of women, because they may show the beginning of the decline of their physical functions, as well as the severity of the event, since more than 50% of them need help to get up, and about 18% need hospitalization due to sequelae. This functional decline has already been indicated as more pronounced in women aged 45–50 years, suggesting a relation with menopause^26^ . The significant onset of accidental falls was also identified in an age group that overlaps with the occurrence of menopause, beginning five years later (50–64 years). However, the variable “menopause” was tested and associated with falls only in the simple regression analysis, and did not maintain an association in the multiple one (adjusted OR), corroborating another study^7^ , in which no association between falls and menopause was established. Thus, age seems to be a more consistent factor than menopause, and the occurrence of falls in younger women points out the need to rethink prevention strategies for the target audience and the appropriate time of application, in order to make them more cost-effective.

Another dissonant point of the current studies and persistent in the models was the presence of hyperuricemia as a factor associated with falls. So much so that, to date, no studies have been found that point to this relation. Hyperuricemia is an established risk factor for metabolic syndrome^27^ and for uric gout, in addition to being closely related to cardiovascular diseases, diabetes, dyslipidemias and metabolic syndrome^28^ . Further studies are therefore needed to understand its association with falls.

Metabolic syndrome is already a factor associated with accidental falls in older people^22^ , and brings with it an increased risk of sensorimotor polyneuropathy, regardless of whether the person had a change in glucose metabolism^29^ . As a consequence, the syndrome may bring muscle hypotrophy, ulcers, change in bone density and functional deficits, leading from gait instabilities to accidental falls. Therefore, polyneuropathy limits the performance of daily activities, causing worse gait patterns and increasing the number of falls^30 - 32^ . In this study, we found association of metabolic syndrome with accidental falls, but it was observed that women did not have an association with glucose metabolism, since diabetes was not associated with accidental falls, as in another study conducted with women aged 50 years or over^7^ and, in a national study, with adults aged over 55 years^5^ .

In relation with bone density, neither osteoporosis nor osteopenia were associated with falls, diverging from a large longitudinal study^7^ in which there was a relation in the last two surveys, with osteoporosis establishing itself as risk factor for falls with advancing age.

Even in relation to chronic-degenerative diseases, no association of heart disease and hypertension with accidental falls was found, which corroborates with an Australian^7^ and a national study^5^ .

The association of depression with falls is often referred to in studies^31 , 32^ , however, unlike what many believe, only its prolonged presence, for more than twelve months, increases the risk of falls, regardless of the use of psychotropic medications^31^ . In general, depression brings changes in the spatiotemporal characteristics of the gait, including a decrease in speed and a change in the time of double support, making it unstable. In depressed people, this lower walking speed was associated with a decrease in the length of the step and an increase in the duration of the walking cycle^32^ .

Sleep disorders — specifically, daytime sleepiness and poor sleep — were associated with the occurrence of falls in 2007. Daytime sleepiness is more common in women, and is associated with increased falls in those who do not use antidepressants^4^ . On the other hand, it should be noted that, the lower the number of hours slept, the higher the risk of falls^3^ . Thus, maintaining a healthy sleep is essential and may be considered prophylactic.

Musculoskeletal pain is also associated with falls, and may be characterized by region. When located in the lower limbs, the association is stronger, possibly due to the mechanics of human gait. In the foot, the chance of older adults falling increases by 138% (OR 2.38; 95%CI 1.62–3.48), and in the hip, by 36% (OR 1.36; 95%CI 1.00–1.84)^24^ . The more severe and lasting it is, the higher the risk of falls. And, finally, there are records that this risk also increases when pain is located in more than one body location^25^ .

## FINAL REMARKS

This study was conducted with a probabilistic and representative sample, which allows the inference or generalization of the results for women from Pindamonhangaba/SP registered in the Family Health Strategy, considering that the FHS covered 33.41% of the city’s population in 2007 and expanded to 39.95% in 2014. Thus, it is possible that other cities, with characteristics similar to Pindamonhangaba, have a similar picture.

On the other hand, this epidemiological study used interviews with a methodological strategy to obtain information, with several questions based on self-reporting; therefore, it is possible that there is memory and information bias. In addition, the clinical investigations carried out, seeking to characterize certain health problems, aimed at screening, not diagnosing. We also reiterate that the study was conducted from cross-sectional data, without longitudinal follow-up of the participating women, not allowing temporal conclusions regarding a possible, but not evaluated, progression of the risk of falls with advancing age.

It was found that accidental falls occur in women aged 50-64 years (middle-aged), therefore, they are not accidents exclusive to elderly; also, hyperuricemia may be a new associated factor. In addition, factors associated with falls have a direct or indirect relation with physical dysfunction, especially of balance and gait. It can be said that falls occur in a context of persistent and dynamic interaction of the factors associated with them. With the exception of age, all factors may be preventable and potentially modifiable with the adoption of healthy life habits and specific clinical treatment, when necessary.
