# Supplementary material for: Accidental falls in middle-aged women
Source: Rev Saude Publica. 2020 Dec 4;54:141. doi: 10.11606/s1518-8787.2020054002579 (PMC7726919; doi:10.11606/s1518-8787.2020054002579)
Supplement: Translation

# Translation: Quedas acidentais em mulheres de meia-idade

**Authors:** Lígia Raquel Ortiz Gomes Stolt, Daniel Vieira Kolish, Maria Regina Alves Cardoso, Clarice Tanaka, Erika Flauzino Silva Vasconcelos, Elaine Cristina Pereira, Máyra Cecilia Dellú, Wendry Maria Paixão Pereira, José Mendes Aldrighi, Ana Carolina Basso Schmitt

**Affiliations:** I Brasil Universidade Federal da Paraíba. Departamento de Fisioterapia. João Pessoa, PB, Brasil; II Brasil Universidade de São Paulo. Faculdade de Medicina. Programa de Pós-Graduação em Ciências da Reabilitação. São Paulo, SP, Brasil; III Brasil Articulab – Ortopedia Moderna Especializada. Fisioterapeuta em reabilitação ortopédica e facilitador de processos de trabalho e desenvolvimento de projetos. São Paulo, SP, Brasil.; IV Brasil Universidade de São Paulo. Faculdade de Saúde Pública. Departamento de Epidemiologia. São Paulo, SP, Brasil; V Brasil Universidade de São Paulo. Faculdade de Medicina. Departamento de Fisioterapia, Fonoaudiologia e Terapia Ocupacional. São Paulo, SP, Brasil; VI Brasil Centro Universitário FUNVIC. Curso de Fisioterapia. Pindamonhangaba, SP, Brasil; VII Brasil Universidade de Taubaté. Departamento de Fisioterapia. Taubaté, SP, Brasil; VIII Brasil Universidade de São Paulo. Faculdade de Saúde Pública. Departamento de Saúde Materno-Infantil. São Paulo, SP, Brasil; IX Brasil Universidade de São Paulo. Faculdade de Medicina. Hospital das Clínicas. Laboratório de Investigação em Fisioterapia. São Paulo, SP, Brasil

**Keywords:** Mulheres, Acidentes por Quedas, Causas Externas, Epidemiologia, Prevalência

## Abstract

**OBJETIVO:**

Estimar a prevalência de quedas acidentais em mulheres e identificar possíveis associações de variáveis sociodemográficas, clínicas e de hábitos de vida com as quedas, em 2007 e 2014.

**MÉTODOS:**

Foram realizados dois estudos transversais, em 2007 e 2014, dentro do Projeto de Saúde de Pindamonhangaba (PROSAPIN), com mulheres com idades variando de 35 a 75 anos. As amostras probabilísticas foram selecionadas dentre as mulheres residentes no município e participantes da Estratégia Saúde da Família. A coleta de dados incluiu: entrevista face a face, exame antropométrico e exame sanguíneo. A variável de desfecho “Sofreu queda nos últimos seis meses?” foi levantada durante a entrevista. Foram estimadas as prevalências de quedas em 2007 e 2014 por ponto e intervalo de confiança de 95% (IC95%). Modelos de regressão logística múltipla foram construídos para identificar a associação das variáveis independentes e a ocorrência de quedas para cada ano a partir da *odds ratio* (OR). Utilizou-se o *software* Stata 14.0 para análise estatística.

**RESULTADOS:**

As prevalências de quedas acidentais foram: 17,6% (IC95% 14,9–20,5) em 2007 e 17,2% (IC95% 14,8–19,8) em 2014. Em 2007 os fatores associados a quedas foram: idade de 50–64 anos (OR = 1,81; IC95% 1,17–2,80), ensino médio (OR = 1,76; IC95% 1,06–2,93), hiperuricemia (OR = 3,74; IC95% 2,17–6,44), depressão (OR = 2,07; IC95% 1,31–3,27), sono ruim (OR = 1,78; IC95% 1,12–2,82) e sonolência diurna (OR = 1,86; IC95% 1,16–2,99). Em 2014 permaneceram: idade de 50–64 anos (OR = 1,64; IC95% 1,04–2,58), hiperuricemia (OR = 1,91; IC95% 1,07–3,43) e depressão (OR = 1,56; IC95% 1,02–2,38), acrescidos da síndrome metabólica (OR = 1,60; IC95% 1,03–2,47) e da dor musculoesquelética (OR = 1,81; IC95% 1,03–3,18).

**CONCLUSÕES:**

As quedas ocorrem de maneira importante em mulheres a partir dos 50 anos, indicando que não são restritas a idosos e que há necessidade de iniciar medidas preventivas mais precocemente. Os dois estudos mostraram magnitudes semelhantes de ocorrência de quedas acidentais e reforçaram sua multifatorialidade. Além disso, a hiperuricemia pode ser um potencial novo fator associado a quedas.

## INTRODUÇÃO

Quedas acidentais são eventos inesperados nos quais as pessoas vão ao chão, assoalho ou a um nível inferior^1 , 2^ . Suas causas são multifatoriais, e a ocorrência de quedas em idosos tem sido associada a: ser mulher^3^ , quase cair^6^ , idade avançada^5^ , déficits visuais^3 , 7 , 8^ , incontinência urinária^6^ , problemas cardiovasculares^6^ , obesidade^7^ , distúrbios do sono^3 , 4 , 6^ , diabetes^6^ , depressão^6^ , entre outros.

Os acidentes por quedas têm sido alvo de estudo constante na comunidade científica, por sua relevância como problema de saúde característico da terceira idade^9^ . Acometem cerca de 30% dos idosos^2 , 4^ , consistindo na principal causa de lesões acidentais nos Estados Unidos^9^ e ocupando a terceira posição entre as causas de mortes acidentais tanto nos Estados Unidos^9^ quanto no Brasil^10^ .

Esse tipo de agravo tem sido extensivamente estudado em idosos^6 , 8 , 11^ . Entretanto, poucos e recentes estudos internacionais apontam que quedas lesionam adultos de todas as idades^9 , 12^ – 35,3% dos que sofrem essas lesões são de meia-idade^9^ . No Brasil, encontramos apenas um estudo^5^ dedicado ao tema, levantando a necessidade de preencher essa lacuna do conhecimento, para aprofundar e expandir estudos relativos a quedas acidentais em populações mais jovens, incluindo pessoas de meia-idade.

Alguns estudos atuais apontam que a prevalência de quedas é variável nas populações, maior entre caucasianos do que em chineses^13^ e caracteristicamente menor em idosos orientais^3 , 9^ , despertando o interesse de pesquisar países onde a população é predominantemente miscigenada, multiétnica e multicultural, como o Brasil.

Quedas são mais comuns no sexo feminino^3^ e apresentam frequência variável de acordo com a região geográfica e idade. Isso fica evidente ao se comparar os resultados de estudo feito na Austrália^4^ , onde 32,2% das idosas caíram, com outro, realizado na China, em que 19,4% das mulheres acima de 45 anos sofreram quedas, e com um terceiro, realizado no Brasil, que constatou que 30,8% das mulheres acima de 55 anos de idade haviam sofrido quedas acidentais^5^ . Destaca-se ainda que mulheres adultas passam por um evento hormonal marcante em suas vidas, a menopausa^7^ , com consequentes distúrbios osteometabólicos, como a osteoporose. Mulheres idosas são as que mais sofrem lesões por quedas^14^ , e, dentre essas lesões em adultos, as fraturas representam 28% do total^9^ .

Com base no exposto, este estudo teve como objetivo estimar a prevalência de quedas acidentais em mulheres, de 35 a 75 anos de idade, além de identificar possíveis associações de variáveis sociodemográficas, clínicas e de hábitos de vida com as quedas, em 2007 e 2014.

## MÉTODOS

### População e Amostra

Em 2007 e 2014, foram realizados dois estudos transversais dentro do Projeto de Saúde de Pindamonhangaba (PROSAPIN), com mulheres de 35 a 65 anos de idade (em 2007), e de 35 a 75 anos (em 2014), participantes da Estratégia Saúde da Família (ESF). Em 2007, a rede assistencial de atenção primária à saúde de Pindamonhangaba/SP compreendia 18 unidades básicas de saúde, com cobertura populacional de 45.537 indivíduos. Em 2014, ampliou para 21 unidades, cobrindo 57.852 pessoas.

A amostra foi probabilística, usando o procedimento de amostragem sistemática, estratificada por idade das mulheres e unidade de saúde, com partilha proporcional. O tamanho da amostra foi calculado a partir da menor prevalência estimada entre as condições clínicas de interesse no estudo. Considerando a prevalência de diabetes em mulheres brasileiras na faixa etária de interesse (9,7%)^15^ , um erro máximo de 3% em 95% das possíveis amostras e ainda ajustando para possíveis perdas (20%), a amostra final para 2007 foi estimada em 875 mulheres, e para 2014 em 1.200 mulheres.

A população de referência foi composta por mulheres residentes em Pindamonhangaba, nas faixas etárias especificadas em 2007 e 2014, cadastradas na ESF. Foram excluídas do estudo aquelas que mudaram para fora do território de abrangência da ESF ou apresentaram incapacidade física e/ou mental para participar da coleta, além das gestantes e falecidas. O estudo foi aprovado pelo Comitê de Ética da Faculdade de Saúde Pública da Universidade de São Paulo/Brasil, Protocolo nº 1776 (2007) e nº 312.957 (2014). Todas as participantes assinaram um termo de consentimento livre e esclarecido antes de sua inclusão no estudo.

### Coleta de Dados

A coleta de dados foi realizada em três etapas: 1) obtenção de informações via questionário aplicado em entrevista face a face, abordando a variável dependente e as variáveis sociodemográficas, clínicas e de hábitos de vida; 2) exame físico e antropometria, realizado por pesquisadores treinados e calibrados; e 3) exame de análise sanguínea.

### Variável de Desfecho

A variável de desfecho foi a ocorrência de quedas, investigada durante a entrevista com a pergunta: “Sofreu queda nos últimos seis meses?”. Em caso positivo, foi questionado: “Quantas?”. A ocorrência de uma única queda caracterizou a mulher como caidora. As quedas foram detalhadas segundo circunstâncias (marcha, transferência), causas (intrínsecas/extrínsecas) e consequências (ajuda para se levantar após a queda, necessidade de hospitalização, ocorrência e localização de fratura pós-queda, e uso de auxiliares para marcha).

### Variáveis Sociodemográficas

Durante a entrevista, foi investigada a idade da pessoa, que posteriormente foi estratificada em três categorias – 35 a 49 anos, adultas; 50 a 64, meia-idade; 65 a 75 anos, idosas –, com pontos de corte visando a comparação com outros estudos. A cor ou raça autodeclarada foi determinada como branca, preta, amarela, parda ou indígena. O estado civil ou marital foi autorrelatado como casada, desquitada ou separada, divorciada, viúva ou solteira. A escolaridade foi considerada a partir do grau de ensino concluído mais elevado (alfabetizada, ensino fundamental, ensino médio, ensino superior, graduação ou pós-graduação, educação de jovens e adultos: supletivo e alfabetização). Investigou-se também a atividade remunerada ou ocupação: trabalho como atividade econômica ou ocupação remunerada em dinheiro, produtos, mercadorias ou benefícios durante ao menos uma hora semanal (exerce ou não exerce atividade remunerada). Por fim, foi levantado o tipo de unidade básica de saúde, classificada em rural, urbana ou mista pelo Cadastro Nacional de Estabelecimentos de Saúde.

### Variáveis Clínicas

Foram investigados durante a entrevista os autorrelatos de menopausa, osteopenia ou osteoporose, cardiopatias e hiperuricemia. Para depressão, foi utilizado o Inventário de Depressão de Beck validado^16^ (pontuação: 0 a 9 = ausência ou sintomas mínimos; 10 a 18 = depressão leve ou disforia; 19 a 29 = depressão moderada; 30 ou mais = depressão severa). Dor musculoesquelética foi levantada por meio do Questionário Nórdico^17^ . Considerou-se a síndrome metabólica conforme a International Diabetes Federation^18^ , sendo necessário apresentar três de cinco itens, incluindo necessariamente o primeiro: 1) cintura > 80 cm; 2) pressão arterial > 130 mmHg para a sistólica, ou 85 mmHg para a diastólica; 3) colesterol alta densidade < 50 mg/dL; 4) triglicérides > 150 mg/dL; e 5) glicemia de jejum > 100 mg/dL. A presença de glicemia de jejum maior ou igual a 126 mg/dL, ou autorrelato de diabetes associado a medicação hipoglicemiante, caracterizou diabetes^18^ . A pressão arterial foi aferida, conforme a 7ª Diretriz Brasileira de Hipertensão Arterial^19^ , utilizando-se um esfigmomanômetro MicroLife de braço, calibrado, digital, automático, testado e validado. Foram realizadas três medidas de pressão arterial no braço esquerdo, com intervalos de um minuto, com a participante sentada. Obteve-se a média de dois valores, descartando-se o discrepante. Consideraram-se hipertensas mulheres com valores de pressão arterial sistólica maiores ou iguais a 140 mmHg, ou diastólica maiores ou iguais a 90 mmHg^19^ , ou aquelas que se autodeclararam hipertensas e utilizavam medicamento anti-hipertensivo.

### Variáveis de Hábitos de Vida

Foram pesquisados os distúrbios do sono: o Índice de Qualidade do Sono de Pittsburgh^20^ avalia a qualidade do sono no último mês, dividido em sete domínios (pontuação de 0 a 21; se maior que 5, é considerado sono ruim); a Escala de Sonolência de Epworth^21^ investiga a presença de sonolência diurna excessiva e mede a possibilidade de cochilar (pontuação de 0 a 24; se maior que 10, há sonolência diurna excessiva).

Como controle de qualidade, coordenadores de campo estiveram presentes nos locais de coleta, e cerca de 10% das entrevistas foram refeitas parcialmente via telefone.

### Análises dos Dados

As prevalências de quedas em 2007 e 2014 foram estimadas por ponto e por intervalo de confiança de 95% (IC95%). Para identificar os fatores associados a quedas, foram usados modelos de regressão logística simples e múltiplos, estimando-se a *odds ratio* (OR) com seus respectivos IC95%. Os modelos múltiplos foram construídos incluindo-se inicialmente todas as variáveis que apareceram como associadas nas análises simples, e, em seguida, todas as demais variáveis foram testadas. Os cálculos estatísticos foram realizados no *software* Stata 14.0.

## RESULTADOS

Participaram do estudo 756 mulheres em 2007, e 998 em 2014 ( Figura 1 ). Em 2007, a média de idade foi de 47,7 anos (desvio-padrão (DP) de 8 anos), e em 2014, 51,9 (DP = 8,8). Em 2007, a amostra foi composta por mulheres com idade de 35–65 anos, das quais apenas uma tinha 65 anos. Em 2014, já havia 13 mulheres nesse estrato etário, das quais algumas participaram desde o início do estudo, e outras foram incluídas em 2014.

**Figura 1:** Amostra estimada e participante do PROSAPIN em 2007 e 2014. Prevalência (IC95%): 2007: 17,6% (14,9–20,5); 2014: 17,2% (14,8–19,8)

A maioria delas foi à escola, tinha companheiro(a) e vivia em bairros atendidos por unidades básicas de saúde urbanas. Cerca de metade das mulheres se autodeclarou branca, e a mesma quantidade afirmou que exercia atividade remunerada.

A prevalência de quedas acidentais foi semelhante nos dois momentos estudados ( Figura 2 ). As quedas foram únicas para 54,8% das mulheres em 2007, e para 72,4% em 2014, e ocorreram principalmente enquanto caminhavam (52,7 e 64,2%, respectivamente). Os principais motivadores de quedas foram tropeços, escorregões e obstáculos. De maneira preocupante, a maioria precisou de ajuda para levantar após a queda, e em 2014 cerca de 18% delas necessitaram ser hospitalizadas ( Figura 3 ). Fraturas aconteceram em cerca de 12% dos casos, principalmente no punho (7,63%, em 2007, e 4,64%, em 2014), e apenas oito pessoas (três em 2007 e cinco em 2014) usavam auxiliares para marcha.

**Figura 2:** Prevalências dos acidentes por quedas nas mulheres do PROSAPIN em 2007 e 2014.

**Figura 3:** Causas (A) e consequências (B) dos acidentes por quedas nas mulheres do PROSAPIN em 2007 e 2014.

As análise de regressão simples antecederam a análise de regressão múltipla e não estabeleceram associações das variáveis menopausa (OR = 1,66; IC95% 1,13–2,43 / OR = 1,60; IC95% 1,10–2,33), osteopenia e osteoporose (OR = 0,96; IC95% 0,29–3,26 / OR = 1,10; IC95% 0,48–2,51), cardiopatias (OR = 1,66; IC95% 0,96–2,87 / OR = 1,76; IC95% 1,10–2,81), diabetes (OR = 1,85; IC95% 1,08–3,17 / OR = 1,39; IC95% 0,92–2,10) e hipertensão (OR = 1,29; IC95% 0,88–1,89 / OR = 1,39; IC95% 0,98–1,97), com as quedas acidentais, em 2007 e 2014. Nas análises de regressão múltipla, os modelos foram distintos para 2007 ( Tabela 1 ) e 2014 ( Tabela 2 ). Ao se observar o peso líquido de cada variável, a hiperuricemia destaca-se nos dois momentos analisados. Idade (50–64 anos), hiperuricemia e depressão estiveram associadas a quedas acidentais tanto em 2007 como em 2014. Já distúrbios do sono e escolaridade (ensino médio) foram importantes apenas em 2007, enquanto dor musculoesquelética e síndrome metabólica apareceram como associadas a quedas somente em 2014.

**Tabela 1:** Resultados dos modelos de regressão simples (OR) múltiplo (OR ajustada) para quedas acidentais e as variáveis sociodemográficas, clínicas e de hábitos de vida, PROSAPIN 2007.

Variáveis sociodemográficas, clínicas e de hábitos de vida	n (%)	OR 2007			OR ajustada 2007		
		OR	IC95%	p	OR	IC95%	p
**Idade** ^a^ **(anos):**							
30–49	62 (13,8)	Ref.	-	-	Ref.	-	-
**50–64**	68 (23,8)	**1,94**	1,32– 2,84	**0,001**	**1,81**	1,17– 2,80	**0,008**
65–75	1 (9,1)	0,62	0,07– 4,94	0,654	-	-	-
**Escolaridade** ^b^							
Alfabetizado, ensino fundamental ou supletivo	86 (15,9)	Ref.	-	-	Ref.	-	-
**Ensino médio**	30 (20,1)	1,33	0,83– 2,11	0,225	**1,76**	1,06– 2,93	**0,029**
Graduação ou Pós-Graduação	6 (42,9)	**3,95**	1,34– 11,70	**0,013**	-	-	-
**Hiperuricemia**	33 (41,3)	**3,90**	2,37– 6,40	**< 0,001**	**3,74**	2,17– 6,44	**< 0,001**
**Depressão**	69 (27,4)	**2,63**	1,79– 3,86	**< 0,001**	**2,07**	1,31– 3,27	**0,002**
Síndrome metabólica	54 (20,1)	**1,72**	1,06– 2,78	**0,027**	-	-	-
**Sono ruim**	83 (24,7)	**2,47**	1,67– 3,65	**< 0,001**	**1,78**	1,12– 2,82	**0,014**
**Sonolência diurna**	45 (26,2)	**2,01**	1,33– 3,02	**0,001**	**1,86**	1,16– 2,99	**0,010**

OR: *odds ratio;* IC95%: intervalo de confiança de 95%.

Nota: Valores com significância estatística estão apresentados em negrito.

Ref.: Categorias de referência para:

^a^Idade (anos): Categoria de referência = (30–49).

^b^Escolaridade: Categoria de referência = alfabetizado, ensino fundamental ou supletivo.

Hiperuricemia, depressão, sono ruim, sonolência diurna, síndrome metabólica e dor musculoesquelética: categoria de referência = ausente.

**Tabela 2:** Resultados dos modelos de regressão simples (OR) múltiplo (OR ajustada) para quedas acidentais e as variáveis sociodemográficas, clínicas e de hábitos de vida, PROSAPIN 2014.

Variáveis sociodemográficas, clínicas e de hábitos de vida	n (%)	OR 2014			OR ajustada 2014		
		OR	IC95%	p	OR	IC95%	p
**Idade** ^**a**^ **(anos)**							
30–49	48 (12,8)	Ref.	-	-	Ref.	-	-
**50–64**	92 (20,7)	**1,79**	1,22– 2,61	**0,003**	**1,64**	1,04– 2,58	**0,033**
65–75	13 (18,6)	1,56	0,79–3,06	0,197	-	-	-
Escolaridade^b^							
Alfabetizado, ensino fundamental ou supletivo	109 (18,4)	Ref.	-	-	-	-	-
Ensino médio	22 (11,1)	**0,55**	0,33– 0,90	**0,017**	-	-	-
Graduação ou Pós-Graduação	9 (18,8)	1,02	0,48– 2,16	0,958	-	-	-
**Hiperuricemia**	26 (28,9)	**2,18**	1,33– 3,57	**0,002**	**1,91**	1,07– 3,43	**0,030**
**Depressão**	92 (20,9)	**1,69**	1,18– 2,40	**0,004**	**1,56**	1,02– 2,38	**0,042**
**Síndrome metabólica**	81 (21,9)	**1,78**	1,18– 2,69	**0,006**	**1,60**	1,03– 2,47	**0,035**
**Dor musculoesquelética**	127 (19,0)	**1,77**	1,13– 2,78	**0,013**	**1,81**	1,03– 3,18	**0,040**
Sono ruim	46 (18,5)	1,13	0,77– 1,65	0,527	-	-	-
Sonolência diurna	37 (17,3)	1,00	0,67– 1,51	0,965	-	-	-

OR: *odds ratio;* IC95%: intervalo de confiança de 95%.

Nota: Valores com significância estatística estão apresentados em negrito.

Ref.: Categorias de referência para:

^a^Idade (anos): Categoria de referência = (30–49).

^b^Escolaridade: Categoria de referência = alfabetizado, ensino fundamental ou supletivo.

Hiperuricemia, depressão, sono ruim, sonolência diurna e síndrome metabólica: categoria de referência = ausente.

## DISCUSSÃO

As prevalências estimadas de quedas acidentais em 2007 e 2014 foram semelhantes, e estão dentro da amplitude de variação de um estudo realizado com pessoas da meia-idade (8,7–31,1%) da Austrália, Holanda, Irlanda e Grã-Bretanha^12^ . Mas, quando comparadas a estudos que envolvem idosos da Austrália^4^ (32,2%), da Inglaterra^8^ (27,3%) e até mesmo do Brasil^11^ (29,1–32,7%), são mais baixas, o que facilita o entendimento de haver maior prevalência de quedas em idades mais avançadas.

No entanto, ao analisarmos os dados de 2007 e 2014 de quedas acidentais segundo a faixa etária, constata-se que as maiores prevalências ocorreram em mulheres de meia-idade (50–64 anos), com valores respectivos de 23,8% e 20,7%. Estas, ao contrário do esperado, foram maiores do que as prevalências estimadas para idosas (65–75 anos), possivelmente devido ao pequeno número de mulheres nessa faixa. Por outro lado, assemelham-se às prevalências encontradas para idosos tailandeses^22^ e chineses^3^ . Ressalta-se ainda que, se as mulheres começam a cair numa idade mais precoce, elas têm maior chance de cair novamente nos anos vindouros, tornando-se caidoras recorrentes. Infelizmente, há uma expectativa de consequências mais graves em quedas recorrentes, resultando em aumento de internações hospitalares e óbitos^13^ .

Quedas trazem consequências avassaladoras, causando cerca de 80% das incapacidades por lesões não intencionais em adultos acima dos 50 anos^14^ provenientes dos países México, Gana, Índia, Rússia e África do Sul – ratificando que não são exclusivas da terceira idade.

Vale ressaltar que quedas causam fraturas específicas de acordo com a faixa etária^23^ . Idosos geralmente caem em baixa velocidade e sobre o quadril, aumentando o risco de fraturas no local. Já adultos de meia-idade movimentam-se em maior velocidade e caem sobre os braços, fraturando em especial úmero ou antebraço distal (punho)^23^ . O fato da maior parte das fraturas ocorridas no presente estudo terem sido no punho condiz com as características de fraturas de pessoas na meia-idade.

A maioria dos fatores associados as quedas de mulheres de meia-idade também se associa as quedas de idosos. Houve mudanças desses fatores na composição do modelo de regressão multivariado entre 2007 e 2014: três deles (50–64 anos, hiperuricemia e depressão) persistiram na composição; sono ruim, sonolência diurna e escolaridade (ensino médio) foram específicos para 2007; e síndrome metabólica e dor musculoesquelética, para 2014.

Dentre os fatores associados à ocorrência de quedas de mulheres adultas, idade^5^ , depressão^7 , 8^ , síndrome metabólica^22^ , dor musculoesquelética^7 , 24 , 25^ , sono ruim^3 , 5^ e sonolência diurna excessiva^4^ já foram associados à ocorrência de quedas de idosos, e de pessoas em outras faixas etárias também.

Idade avançada é frequentemente associada à ocorrência de quedas em ambos os sexos^8^ . Entretanto, constatou-se associação marcante da idade de 50–64 anos com a ocorrência de quedas, revelando que houve maior prevalência em mulheres de meia-idade do que em adultas mais jovens. Portanto, quedas acidentais não constituem agravos de saúde exclusivos de pessoas idosas, apesar da grande quantidade de estudos realizados com essa população.

A ocorrência de quedas de pessoas na meia-idade, portanto antes da senescência, tem sido apontada em alguns estudos recentes^7 , 9^ , os quais registraram alta taxa de incidência de quedas (42,8%, IC95% 34,9–50,8) de mulheres nessa faixa etária. Um estudo^12^ reforça a ocorrência de quedas na meia-idade, especialmente de mulheres. No Brasil, um único estudo realizado com pessoas de 55 e mais anos^5^ encontrou associação entre quedas e idade, porém avançada (igual ou superior a 65 anos).

Portanto constatamos que mulheres de 50 a 64 anos, ainda na meia-idade, caem de maneira significante, quebrando o paradigma de quedas acidentais serem restritas a pessoas idosas – fato não publicado ainda em estudos nacionais. Além disso, evidencia-se que quedas são marcantes na vida da mulher, pois podem transparecer o início do declínio de suas funções físicas, bem como se nota a gravidade do evento, pois mais de 50% delas precisam de ajuda para se levantar, e cerca de 18% necessitam de hospitalização devido a sequelas. Esse declínio funcional já foi apontado como mais acentuado em mulheres de 45–50 anos, sugerindo relação com a menopausa^26^ . Também se identificou o início significativo de quedas acidentais num estrato etário que se sobrepõe à ocorrência da menopausa, com início cinco anos mais tarde (50–64 anos). Entretanto, a variável “menopausa” foi testada e associada a quedas apenas na análise de regressão simples, e não manteve associação na múltipla (OR ajustada), corroborando outro estudo^7^ , em que não foi estabelecida associação entre quedas e menopausa. Assim, idade parece ser fator mais consistente que menopausa, e a ocorrência de quedas de mulheres mais novas aponta a necessidade de repensar as estratégias de prevenção para o público-alvo e o momento adequado de aplicação, a fim de torná-las mais custo-efetivas.

Outro ponto dissonante dos estudos atuais e persistente nos modelos foi a presença da hiperuricemia como fator associado a quedas. Tanto é que, até o momento, não foram encontrados estudos que apontem essa relação. A hiperuricemia é fator de risco estabelecido para síndrome metabólica^27^ e para gota úrica, além de ser intimamente relacionada a doenças cardiovasculares, diabetes, dislipidemias e a própria síndrome metabólica^28^ . São necessários, portanto, estudos mais aprofundados para compreender sua associação com quedas.

A síndrome metabólica já é um fator associado a quedas acidentais de idosos^22^ , e traz consigo um risco aumentado de polineuropatia sensório-motora, independentemente de a pessoa apresentar alteração no metabolismo da glicose^29^ . Como consequência, a síndrome pode trazer hipotrofia muscular, úlceras, mudança na densidade óssea e déficits funcionais, levando desde a instabilidade na marcha até quedas acidentais. Portanto, a polineuropatia limita a realização de atividades diárias, ocasionando piores padrões de marcha e aumentando o número de quedas^30^ . No presente estudo, encontrou-se associação de síndrome metabólica com quedas acidentais, mas se observou que as mulheres não apresentaram associação com o metabolismo da glicose, visto que a diabetes não foi associada a quedas acidentais, assim como em outro estudo realizado com mulheres a partir de 50 anos^7^ e em um nacional, com adultos a partir de 55 anos^5^ .

Em relação à densidade óssea, nem a osteoporose nem a osteopenia foram associadas a quedas, divergindo de um grande estudo longitudinal^7^ em que houve relação nos dois últimos *surveys* , com a osteoporose se estabelecendo como fator de risco para quedas com o avanço da idade.

Ainda em relação a doenças crônico-degenerativas, não foi encontrada qualquer associação de cardiopatias e hipertensão com quedas acidentais, o que corrobora o estudo australiano^7^ e outro nacional^5^ .

A associação de depressão a quedas é com frequência referida em trabalhos^31 , 32^ , mas, diferente do que muitos acreditam, apenas sua presença prolongada, por mais de doze meses, aumenta o risco de quedas, independente do uso de medicações psicotrópicas^31^ . De maneira geral, a depressão traz modificações nas características espaço-temporais da marcha, incluindo diminuição de velocidade e alteração do tempo de suporte duplo, tornando-a instável. Nos depressivos, essa menor velocidade de marcha foi associada a diminuição do comprimento do passo e aumento da duração do ciclo da marcha^32^ .

Distúrbios do sono – especificamente, sonolência diurna excessiva e sono ruim – foram associados à ocorrência de quedas em 2007. A sonolência diurna é mais frequente em mulheres, e está associada ao aumento de quedas daquelas que não usam antidepressivos^4^ . Por outro lado, destaca-se que, quanto menor for o número de horas dormidas, maior é o risco de quedas^3^ . Assim, a manutenção de um sono saudável é essencial e pode ser considerada profilática.

Dor musculoesquelética também é associada a quedas, e pode ser caracterizada por região. Quando localizada nos membros inferiores, a associação é mais forte, possivelmente devido à mecânica da marcha humana. No pé, aumenta em 138% a chance de idosos caírem (OR 2,38; IC95% 1,62–3,48), e, no quadril, em 36% (OR 1,36; IC95% 1,00–1,84)^24^ . Quanto mais severa e duradoura for, maior será o risco de quedas. E, por fim, há registros de que esse risco também aumenta quando a dor é localizada em mais de um local corporal^25^ .

## CONSIDERAÇÕES FINAIS

Este estudo foi conduzido com uma amostra probabilística e representativa, que possibilita a inferência ou generalização dos resultados para as mulheres de Pindamonhangaba/SP cadastradas na Estratégia de Saúde da Família, considerando que a ESF cobria 33,41% da população da cidade em 2007 e expandiu para 39,95% em 2014. Assim, é possível que outras cidades, com características semelhantes a Pindamonhangaba, apresentem quadro similar.

Por outro lado, esse estudo epidemiológico utilizou entrevistas como uma estratégia metodológica para obter informações, com realização de diversas questões baseadas em autorrelato; portanto, é possível haver viés de memória e informação. Além disso, as investigações clínicas realizadas, procurando caracterizar determinados agravos de saúde, tiveram objetivo de rastreamento, e não de diagnóstico. Reitera-se também que o estudo foi realizado a partir de dados transversais, sem acompanhamento longitudinal das mulheres participantes, não permitindo ilações temporais quanto a uma possível, porém não avaliada, progressão do risco de quedas com o avanço da idade.

Constata-se que quedas acidentais ocorrem em mulheres de 50–64 anos (meia-idade), portanto não são acidentes exclusivos de idosos, e que a hiperuricemia pode ser um novo fator associado. Além disso, os fatores associados a quedas têm relação direta ou indireta com a disfunção física, especialmente do equilíbrio e marcha. Pode-se dizer que quedas ocorrem num contexto de interação persistente e dinâmica dos fatores a elas associados. Com exceção da idade, todos os fatores podem ser preveníveis e potencialmente modificáveis com adoção de hábitos de vida saudáveis e tratamento clínico específico, quando necessário.
